# Phosphoproteomics Unravel HBV Triggered Rewiring of Host Phosphosignaling Events

**DOI:** 10.3390/ijms23095127

**Published:** 2022-05-04

**Authors:** ZiJie Lim, Nur Khairiah Binte Mohd-Ismail, Evelyn Png, Ching Wooen Sze, Qifeng Lin, Wanjin Hong, Seng Gee Lim, Yee-Joo Tan, Jayantha Gunaratne

**Affiliations:** 1 Institute of Molecular and Cell Biology, Agency for Science Technology and Research, Singapore 138673, Singapore; limz@imcb.a-star.edu.sg (Z.L.); dbslinq@nus.edu.sg (Q.L.); mcbhwj@imcb.a-star.edu.sg (W.H.); 2 Infectious Diseases Translational Research Programme, Department of Microbiology and Immunology, Yong Loo Lin School of Medicine, National University Health System, National University of Singapore, Singapore 117545, Singapore; khairiah@gmail.com (N.K.B.M.-I.); evelynpngys@yahoo.com (E.P.); 3 Department of Oral and Craniofacial Molecular Biology, Philips Institute for Oral Health Research, Virginia Commonwealth University, Richmond, VA 23298, USA; cwsze@vcu.edu; 4 Division of Gastroenterology and Hepatology, Department of Medicine, National University Hospital, University Medicine Cluster, National University Health System, Singapore 119228, Singapore; mdclimsg@nus.edu.sg; 5 Department of Anatomy, Yong Loo Lin School of Medicine, National University Health System, National University of Singapore, Singapore 117594, Singapore

**Keywords:** hepatitis B virus, phosphoproteomics, phosphosignaling, kinases, kinase inhibitor, kinome

## Abstract

Hepatitis B virus (HBV) infection persists as a major global health problem despite the availability of HBV vaccines for disease prevention. However, vaccination rates remains low in some regions of the world, driving the need for novel strategies to minimise infections and prevent disease progression. Thus, understanding of perturbed molecular signaling events during early phases of HBV infection is required. Phosphosignaling is known to be involved in the HBV infection processes, yet systems-level changes in phosphosignaling pathways in the host during infection remain unclear. To this end, we performed phosphoproteome profiling on HBV-infected HepG2-NTCP cells. Our results showed that HBV infection drastically altered the host phosphoproteome and its associated proteins, including kinases. Computational analysis of this phosphoproteome revealed dysregulation of the pathways involved in immune responses, cell cycle processes, and RNA processing during HBV infection. Kinase Substrate Enrichment Analysis (KSEA) identified the dysregulated activities of important kinases, including those from CMGC (CDK, MAPK, GSK, and CLK), AGC (protein kinase A, G, and C), and TK (Tyrosine Kinase) families. Of note, the inhibition of CLKs significantly reduced HBV infection in HepG2-NTCP cells. In all, our study unravelled the aberrated phosphosignaling pathways and the associated kinases, presenting potential entry points for developing novel therapeutic strategies for HBV treatment.

## 1. Introduction

Hepatitis B virus (HBV) infection is the leading cause for liver fibrosis, cirrhosis, and hepatocellular carcinoma (HCC) [1]. Despite the development of prophylactic vaccinations against HBV, over 300 million people are still chronically infected with HBV and afflicted with accompanying liver diseases, elevating the financial burden on the global healthcare system [2]. Current HBV therapeutics include interferons (IFN) and nucleoside/nucleotide analogue treatment. However, as such treatments are unable to effectively eliminate the viral genome from infected hepatocytes, recurrence of infection is common when treatments are discontinued. To address the urgent need for novel therapeutic approaches in HBV, in-depth knowledge of the molecular signaling and pathways associated with HBV infection is required.

The HBV lifecycle is a multistep process involving both viral and host factors. To initiate virus entry, the large surface HBV protein first interacts with the host sodium taurocholate co-transporting polypeptide (NTCP) membrane transporter [3]. The HBV genome, initially existing as a relaxed circular double-stranded DNA (rcDNA), is subsequently transported to the host nucleus. At this point, the HBV DNA can either be integrated into the host genome, or converted into covalently closed circular DNA (cccDNA) through host DNA repair mechanisms [4]. Through subsequent interactions with host histones and HBV core protein, the cccDNA is organised into a mini-chromosomes that serves as a stable transcriptional template for the synthesis of viral proteins and progeny virions [5]. Adding further complexity, viral and host factors involved in the infection process are also regulated through post-translational modifications (PTMs) such as ubiquitinations, phosphorylations, and methylations (reviewed in [6]). Among these, protein phosphorylation is increasingly recognised as an important PTM that influences the HBV lifecycle. The phosphorylation of Hepatitis B core protein (HBcAg) by kinases such as the serine-arginine rich protein kinase 1/2 (SRPK1/2) and Casein Kinase 2 (CK2) were reported to be vital for protein localisation, pgRNA encapsidation, viral DNA synthesis, and virion maturation and secretion [7,8,9,10]. On a systemic level, protein phosphorylation is responsible for regulating host molecular signaling pathways in response to external or internal stimuli. The interplay and dynamics of phosphosignaling events, such as the TLR2/MyD88/NF-κβ, HIPPO, and cGAS-STING signaling pathways reportedly regulate the outcome of HBV infection [11,12]. Phosphosignaling events are also involved in the development of HBV-associated cirrhosis and hepatocellular carcinoma, which are the main causes of liver morbidity and mortality in chronic HBV patients [1]. For instance, the HBV X protein (HBx) was shown to facilitate HCC development through the activation of phosphosignaling pathways such as the TGF-β signaling pathway (reviewed in [13]). 

The above studies clearly demonstrate that phosphorylation is one of the vital processes in the regulation of HBV infection, playing key roles ranging from regulating the immune responses to promoting HCC progression. However, the dynamics of the global phosphorylation landscape during HBV infection remains poorly understood. Mass spectrometry-based phosphoproteomics is a forefront technology for the unbiased identification and quantification of system-wide phosphoproteome changes. In combination with advanced computational approaches, the rewiring of host signaling pathways during HBV infection can be identified. Additionally, well-annotated protein phosphorylation profiles and state-of-the-art bioinformatics can be used to identify kinome dynamics during HBV infection [14]. Previously, such approaches have been successfully employed to study the effects of phosphosignaling network changes in HBV-associated HCC [15]. However, the understanding about modulations of the phosphorylation landscape during the initial phase of HBV infection remains enigmatic. Here, we report the identification of dysregulated molecular signaling events in early-stage HBV infection through a comprehensive phosphoproteomics analysis of HBV-infected HepG2-hNTCP-C4 cells (HepG2-NTCP). Our analysis revealed the rewiring of the host kinome and phosphosignaling events at seven days post-HBV infection with key kinases as potential therapeutic targets for HBV treatment.

## 2. Results

### 2.1. Altered Phosphoproteome Landscape during HBV-Infection 

**Figure 1:**
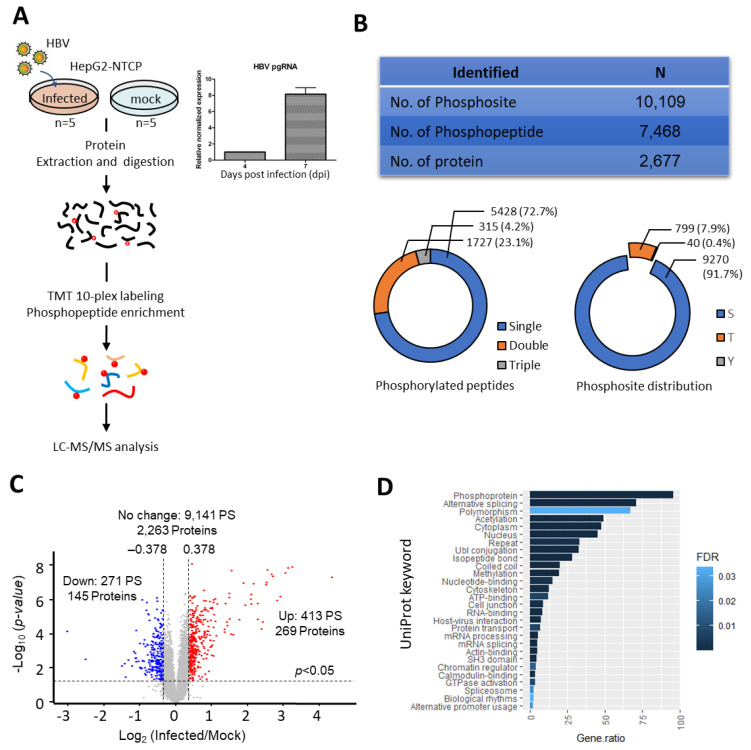
Dysregulation of host phosphoproteome detected during HBV infection (**A**) Left Panel: Schematic representation of TMT-based mass spectrometry strategy for phosphoproteome profiling of HBV infection. Phosphoproteome profiling was performed on HepG2-NTCP cell line infected with HBV at seven days post infection (dpi) at 3000 MOI. Mock infected cells were used as controls. Five biological replicates for both mock and infected HepG2-NTCP. Right panel: High pgRNA expression level detected at seven dpi. (**B**) A total of 10,109 phosphosites were identified, mapping to 7468 phosphopeptides, and 2677 proteins. The pie charts depict the phosphopeptide and phosphosites distributions (**C**) Volcano-plot of phosphopeptides with up- and down-regulated phosphorylation. Phosphopeptides were considered differentially regulated when fold change of HBV/Mock is >1.3 [upregulated, Log2(FC) > 0.378] and <0.769 [downregulated, Log2(FC) < −0.378] in addition to *p-*value <0.05, as determined by the Student’s *t*-test (**D**) UniProt keywords linked to proteins with deregulated phosphorylation. Only keywords with FDR <0.05 are shown.

To understand the rewiring of phosphosignaling events during HBV infection, a global phosphoproteomics analysis was performed using HBV-infected HepG2-NTCP cells at seven dpi. Mock-infected HepG2-NTCP cells at seven dpi were used as controls. To identify and quantify global phosphorylation changes, we employed the tandem mass tag (TMT)-based phosphoproteomics approach (workflow shown in Figure 1A). Based on the HBV pgRNA levels, we observed a MOI of 3000 to be sufficient for obtaining a measurable level of infection at seven dpi. (Figure 1A and Supplementary Figure S1). Through this analysis, we identified 10,109 phosphosites, of which 9825 sites are with high-quality quantification. These sites were derived from 7468 phosphopeptides (2677 phosphoproteins). The majority of these peptides (73%) harbor a single phosphosites, while 23% and 4% have double and triple site-occupancies, respectively (Figure 1B). Of the quantified phosphosites, 72% have a localisation probability of more than 0.98, among which 92%, 8%, and 0.4% of the phosphosites are pSerine(S), pThreonine(T), and pTyrosine(Y), respectively (Figure 1B). In comparison to the Phosphosite Plus database [16], 29% of our identified phosphosites were novel and unique to our dataset.

Unsupervised hierarchical clustering and Principal Component Analysis (PCA) analysis showed tight clustering among the replicates while forming distinct clusters between HBV and mock infected replicates (Supplementary Figure S2A,B), thereby indicating the high reproducibility of our dataset. Using the criteria of TMT ratio >1.3 (up-regulation) and <0.769 (down-regulation) with a *p*-value <0.05, we identified 413 phosphosites on 340 phosphopeptides (269 proteins) with increased abundance and 271 phosphosites on 198 phosphopeptides (145 proteins) with decreased abundances (Figure 1C and Supplementary Table S1), thereby implying substantial changes to the host phosphorylation landscape at seven dpi. Substantially higher number of phosphorylation events (413) as compared to dephosphorylation (273) were observed, suggesting a global enhancement of kinase activity during infection. Based on the annotations from the UniProt database, the proteins identified with altered phosphorylation changes are known phosphoproteins, demonstrating high phosphopeptide recovery efficiency. Additionally, some of them are associated with host–virus interactions, confirming the suitability of this model in studying the host response to HBV infection (Figure 1D).

### 2.2. Dysregulation of Biological Processes during HBV Infection

**Figure 2:**
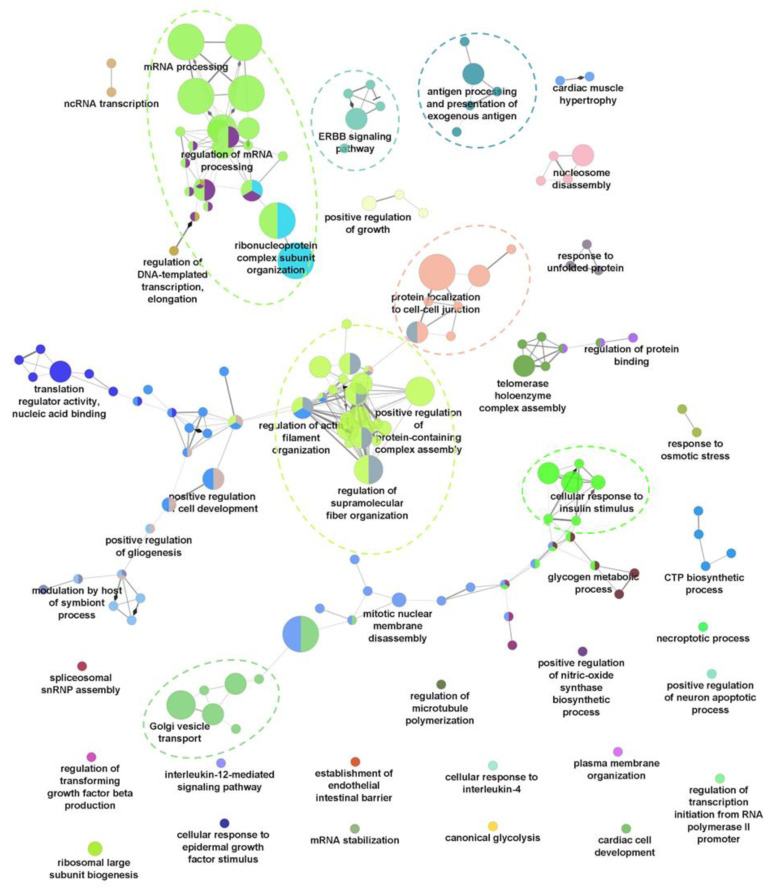
Gene Ontology Biological Process (GO-BP) analysis of proteins with upregulated phosphorylation using ClueGO. Main ClueGo clusters denoted by dashed line.

To evaluate the global changes of biological processes upon seven dpi, a Gene Ontology Biological Process (GO-BP) functional annotation of the differentially regulated phosphoproteins was performed using ClueGO [17]. Proteins with increased phosphorylation were shown to be associated with 159 representative GO-BP terms, forming 44 ClueGO clusters based on kappa scoring (Figure 2). Among these, seven main ClueGO clusters containing a high significance of GO-BP terms were observed (Supplementary Figure S4). The main clusters include RNA processing, regulation of supramolecular fibre organisation, golgi vesicle transport, cellular response to insulin stimulus, protein localisation to cell–cell junctions, ERBB signaling pathway, and exogenous antigen processing and presentation (Figure 2). In contrast, the functional annotation of proteins with decreased phosphorylation was only assigned to 20 representative GO-BP terms (Supplementary Figure S3). Some of the significant (*p*-value < 0.05) GO-BP terms associated with this group of proteins include the cellular response to interleukin-4 (IL-4), positive regulation of histone modification, and membrane assembly (Supplementary Figure S3). These results suggest that, HBV infection at seven dpi causes significant changes to host biological processes, especially through the upregulation of protein phosphorylation, which is likely owed to the respective kinase activation.

The activation and regulation of the innate host immune system are among the first host defences against viral infection. To determine whether these proteins with altered phosphorylation are involved in the host immune responses, a limited GO enrichment assay was performed against GO terms related to immune system processes (ISP). Of the 356 proteins identified with altered phosphorylation, 74 were annotated to 45 representative GO-ISP terms such as granulocyte activation and neutrophil degranulation and activation, among others (Supplementary Figure S5C). The heatmap depicting the normalised abundance (z-score) of these phosphosites is shown in Supplementary Figure S5D. Based on kappa score grouping, these 45 representative GO-ISP terms can be grouped into seven clusters (Supplementary Figure S5A). These clusters were represented by GO terms that are linked to granulocyte differentiation and activation, B-cell proliferation, antigen processing and presentation, hematopoietic stem cell differentiation, activation of innate immune responses, and defence responses to a virus (Supplementary Figure S5A). Among these, GO-ISP terms that are grouped in granulocyte activation were statistically significant, with an adjusted *p*-value of <0.05 (Supplementary Figure S5B).

### 2.3. HBV Infection-Induced Host Kinome 

**Figure 3:**
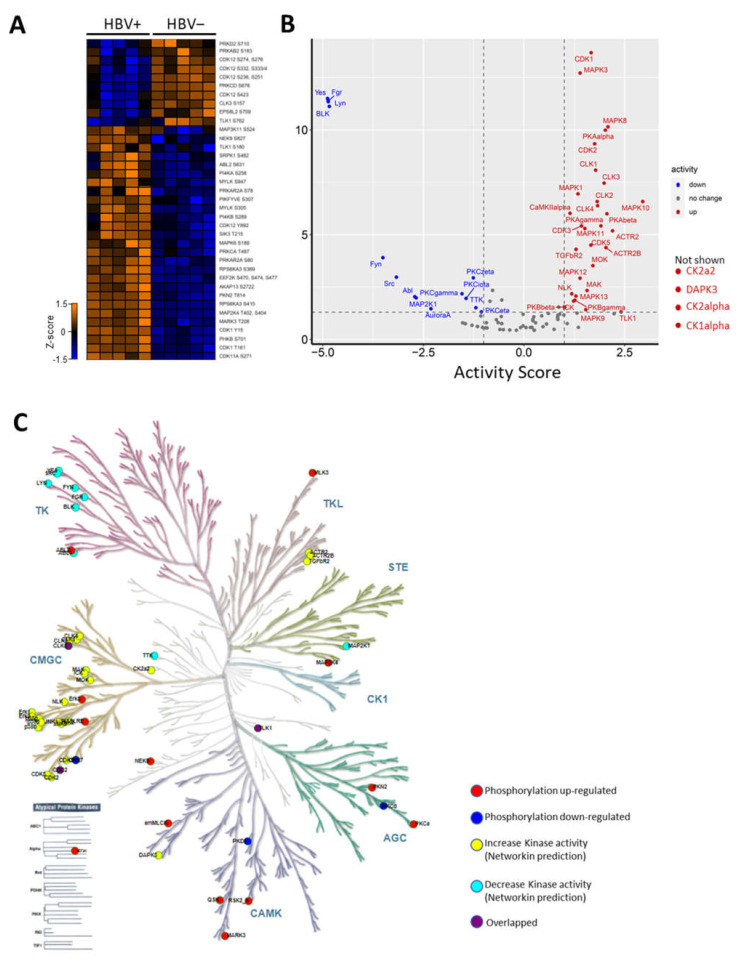
Host kinome alteration during HBV infection (**A**) Heatmap (z-score) illustration of identified kinases with deregulated phosphorylations (**B**) Kinase activity plot depicting estimated kinase activities during HBV infection. Kinases with enhanced activities are labelled in red, while kinases with decreased activities are in blue. Kinases with no changes in activity are not labelled. CK2a2, DAPK3, CK2alpha, and CK1alpha are not shown due to their off-the-chart activity scores. (**C**) Kinase phylogenetic tree depicting kinase families deregulated during HBV infection.

To investigate how the kinases were affected during HBV infection, we examined the kinases quantified in our data and found that the phosphorylation of 28 kinases were altered during HBV infection (Figure 3A). Of these, the upregulation of serine/arginine-rich protein specific kinase (SRPK1) phosphorylation at ser482 was a particularly intriguing novel phosphosite (refer to Supplementary Figure S6 for MS/MS spectra). Moreover, SRPK1 regulates the phosphorylation of the HBcAg C-terminal domain (CTD) region, which is a process known to be vital in the HBV lifecycle [18].

Next, the changes in kinase activities during HBV infection were estimated based on the kinase–substrate relationship using KSEA through NetworKIN [14]. Based on this prediction, the activities of 34 kinases, including CDK1/2, CK1, CK2, and MAPKs were predicted to be upregulated. In contrast, the activities of only 14 kinases, such as Fyn, Src, and PKCs, were predicted to be downregulated during HBV infection (Figure 3B). To gain an overview of the dysregulation of the host kinome during infection, kinases with observed modulated activities and altered phosphorylation were mapped to the kinase phylogenetic tree using KinMap [19]. This mapping indicated that most of the kinases with augmented activities belong to the CMGC (CDK, MAPK, GSK, and CLK) kinase group (Figure 3C). On the other hand, kinases with impaired activities during infection mainly belonged to the TK (Tyrosine Kinase) group (Figure 3C). Together, these results revealed drastic changes in the host kinome that could trigger the dysregulation of key host regulatory pathways during HBV infection. 

### 2.4. Rewiring of Host Phosphosignaling Pathway during HBV Infection

**Figure 4:**
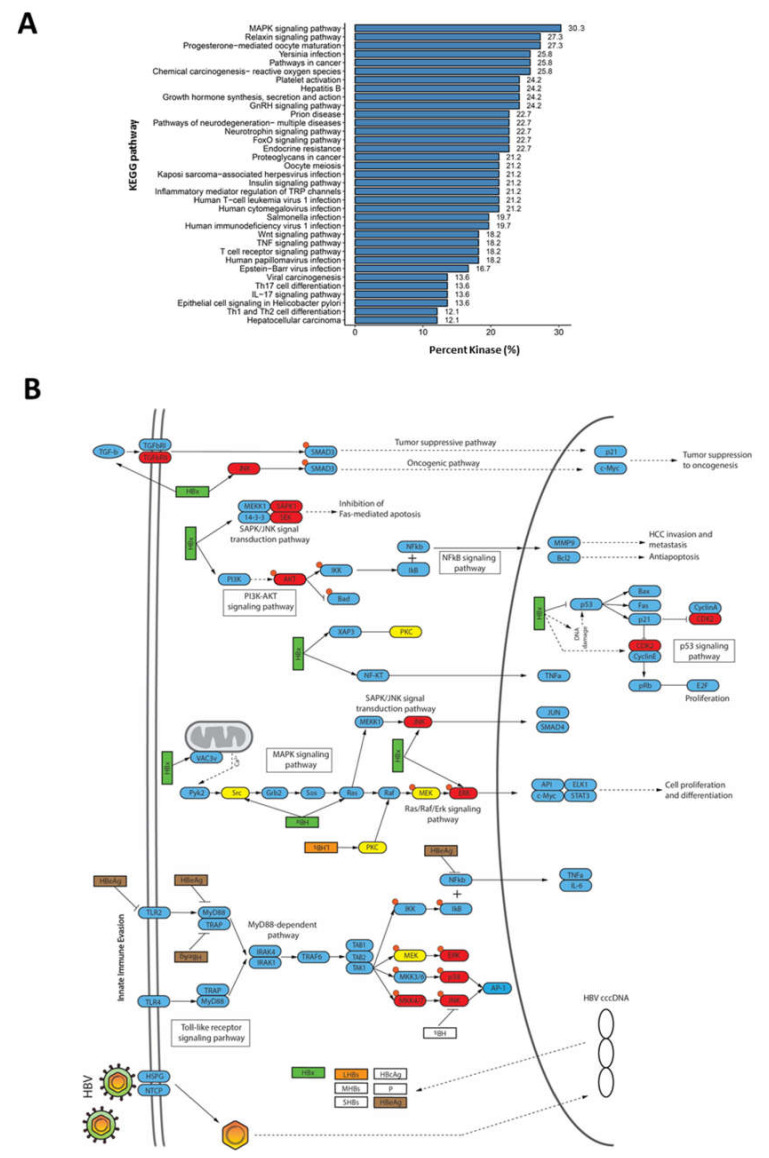
Involvement of dysregulated kinases during HBV infection in various signaling pathways (**A**) KEGG pathway terms associated with kinases deregulated during HBV infection. (**B**) Mapping of kinases with deregulated activities to pathways involved in HBV infection. Nodes in red depict kinases with enhance activities, while nodes in yellow are kinases with lower activities during HBV infection.

To further determine which host pathways are affected during HBV infection, the annotation of dysregulated kinases against the KEGG pathway database revealed that the kinases are involved in several signaling pathways. Broadly, we observed two main associated functional pathway categories: (i) host response to pathogen infection, which includes Yersina infection, Hepatitis B, platelet activation, herpesvirus infection, and Epstein-Barr virus infection; and (ii) tumour progression and cancer-related pathways, including HCC and viral carcinogenesis (Figure 4A,B). 

Sixteen (24%) of the dysregulated kinases identified in this study were mapped to the KEGG pathway database entry, “Hepatitis B” (entry: map05161), which is a collection of signaling pathways involved in regulating HBV infection (Figure 4B). Several kinases, such as TGFβRII, JNK, CKD1/2, and AKT kinases, were associated with signaling pathways that modulate oncogenesis, apoptosis, cell proliferation, and HCC invasion and metastasis. These signaling pathways include TGF-β, PI3K-AKT, SAPK/JNK, and MAPK signaling pathways (Figure 4B). Several kinases, such as p38, ERK, AP-1, and MKK4/7, were mapped to the TLR/MyD88-dependent pathway, which is known to regulate the host innate immune responses to viral infection (Figure 4B). Interestingly, the majority of these were predicted to have higher kinase activities, which suggest that the host immune response is activated through the TLR/MyD88-dependent pathway during HBV infection. This is consistent with several reports where HBV can elicit a limited host innate immune responses [20]. Taken together, our observations confirm that HBV infection rewires diverse host signaling pathways during infection.

### 2.5. Assessing Deregulated Kinases as Therapeutic Targets for HBV 

**Figure 5:**
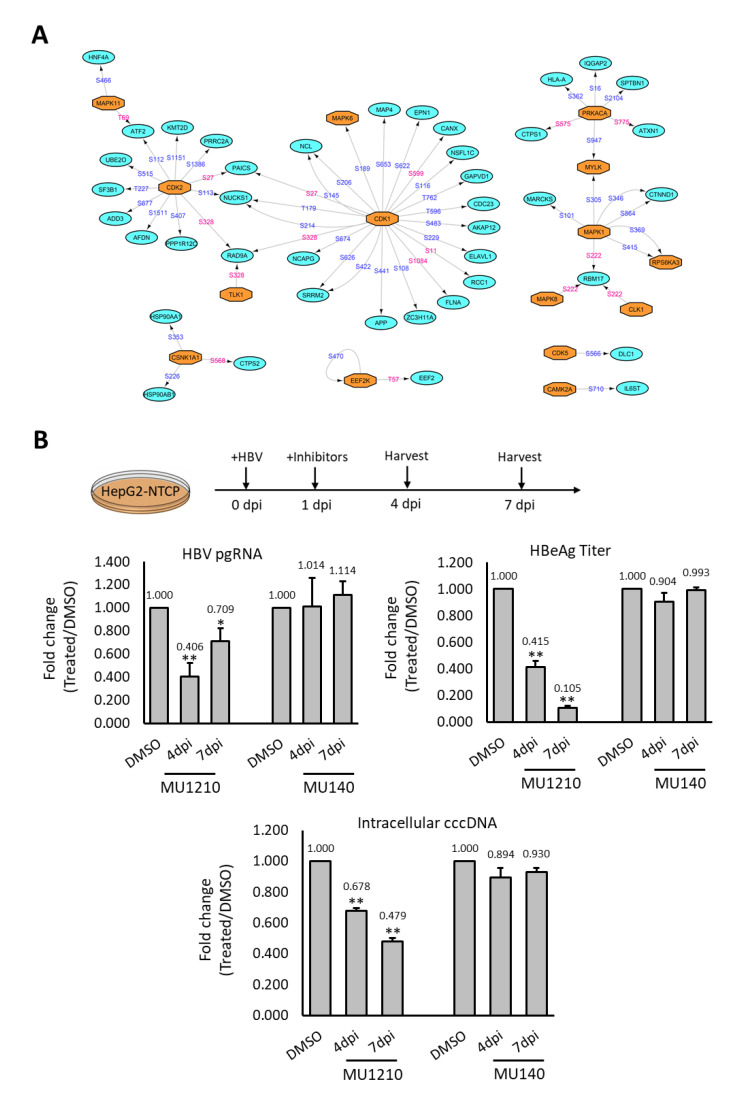
Inhibition of HBV responsive kinase decreases HBV infection (**A**) Kinase–substrate network of the deregulated phosphorylation events observed experimentally. Phosphosites labelled in blue are reported while those labelled in pink are novel phosphosites. (**B**) Effects of CLKs inhibition on HBV infection in HepG2-NTCP cells. HepG2-NTCP was treated with 1 µM of MU1210 (inhibitor) and 1 µM of MU140 (control) during HBV infection. PgRNA level, intracellular cccDNA level, and HBeAg secretion were used as indicators for HBV infection efficiency. Results shown were derived from three biological replicates and *p*-value was calculated using the Student’s *t*-test (two-tailed, * *p*-value < 0.05, ** *p*-value < 0.01).

To decipher how the phosphorylation landscape is deregulated during HBV infection, we constructed a phosphorylation network between the kinases that are predicted and experimentally observed in this study, with their corresponding dysregulated substrates (Figure 5A). This network was constructed, using only experimentally observed substrates and reported kinase–substrate pairs, resulting in a much smaller kinase–substrate network. This was done to facilitate identification of potential kinase therapeutic targets. The constructed phosphorylation network revealed two kinase–substrate network hubs surrounding CDK1 and CDK2, where several substrates with altered phosphosites were observed. Additionally, several MAP kinases and their substrates were also represented in the kinase–substrate network (Figure 5A). This reconstructed network suggests that kinases such as CDK1, CDK2, and MAP kinases could play vital roles in regulating HBV infection through the modulation of phosphosignaling pathways, which is consistent with their reported roles during HBV infection [21,22]. 

Although the CLKs did not form any key network hub, their activation in HBV infection is novel and has not been studied before. Thus, the effect of CLKs inhibitors on HBV was tested with pgRNA expression, intracellular cccDNA, and HBeAg ELISA as indicators for HBV infection efficiency [23,24]. The inhibition of CK2 by DMAT1 was reported to decrease HBV infection, and it was included here as a positive control [25]. As expected, DMAT1 treatment showed a reduction of pgRNA transcription when assayed at both four and seven dpi (Supplementary Figure S8). The treatment of HepG2-NTCP with CLKs inhibitor, MU1210, during HBV infection led to a reduction in the pgRNA level at both four dpi and seven dpi, while no such reduction was observed with MU140, a structural analogue of MU1210 functioning as a negative control (Figure 5B). Similarly, a reduction in intracellular cccDNA and HBeAg secretion levels was also observed (Figure 5B). These observations suggested that CLKs could be plausible candidate targets and their inhibitors can be further evaluated for the development of effective therapies for HBV infection.

## 3. Discussion

In this study, we unravelled the dysregulation of the host kinome and its associated key phosphosignaling events during HBV infection using advanced systemic phosphoproteomics analysis. To our knowledge, this is the first systems-wide study of the host phosphosignaling changes at as early as seven days post-HBV infection. 

Here, we observed several kinases with altered activities that might be attributed to host–virus interactions during HBV infection, suggesting that some could be candidate therapeutic targets for HBV infection. Among them, kinases belonging to the CMGC kinase family, which include CDKs, MAPKs, GSKs, and CLKs, were predict to be activated during HBV infection (Figure 3B). Intriguingly, when compared to a similar study performed on HBV-associated HCC, the enhanced activity of the CMGC kinase family appeared to be specific to early-stage HBV infection [15]. This suggests that CMGC kinases may play dominant roles in regulating early phases of HBV infection. Traditionally, kinases in the CMGC family are involved in diverse biological processes including RNA processing and transcription [26]. 

Our analysis also revealed that a significant portion of proteins with altered phosphorylation were involved in similar biological processes (Figure 2A). Recent evidence has increasingly showed that the interplay between CMGC kinases (especially CLKs and SRPKs) and their roles in the phosphorylation of SR proteins could be implicated in viral replication through the regulation of RNA transcription/splicing, mRNA stability/export, nuclear transport, and protein translation (reviewed in [27]). The phosphorylation of HBcAg (SR domain containing viral protein) at the CTD by SRPK1/2 kinases is thought to be crucial for regulating the affinity between HBcAg and pgRNA, a key step in pgRNA packaging and encapsidation [18]. In our study, we observed the deregulation of SRPK1 phosphorylation at a novel phosphorylation site, Ser482. While SRPK1 is currently understood to be activated by Casein Kinase 2 (CK2) through phosphorylation at Ser51 and Ser555 [28], the effects of Ser482 phosphorylation on the function of SRPK1 remains convoluted. As SRPK1 phosphopeptides containing Ser51 and Ser555 were not detected in our analysis (likely due to stochastic sampling in the MS), the activity status of SRPK1 during HBV infection remains to be concluded. Hence, further studies will be needed to determine whether phosphorylation of SRPK1 at Ser482 has a unique or additive role in regulating HBV infection.

The increased activities of MAPK kinases were also predicted during HBV infection, suggesting the upregulation or activation of the MAPK signaling pathway. This observation is consistent with previous reports where the Ras/Raf/MEK/ERK signaling cascade has been shown to be activated when truncated middle surface antigens [MHBs(t)] were overexpressed in liver cell lines [29]. This was demonstrated to be involved in promoting cell survival and cell cycle deregulation, thereby possibly playing a role in HBV-associated HCC progression [30]. Furthermore, the increased kinase activity of CDK1 observed in our study corroborates with reports suggesting the activation of cyclin B1-CDK1 kinase activity in HBxAg expressing HepG2 cells, will result in cell cycle deregulation through G2/M arrest [31]. However, the mechanism and reasons underlying the induction of cell cycle arrest by HBxAg is still unclear, although a recent report suggested that this might be required to facilitate efficient viral replication [32]. The enhanced activities of kinases involved in the cell cycle support the observation about the increased phosphorylation of their substrates being involved in the cell cycle/division during HBV infection (Figure 2A). 

The innate immune responses to pathogen infection is an integral part of the host defence mechanism. Toll-like receptors (TLRs) play crucial roles in the innate immune response through recognition of pathogen-associated molecular patterns (PAMP) and the subsequent initiation of signaling pathways, thereby causing the ultimate activation of NF-κB transcription factors and IFN response factors (IRFs) [33]. However, since HBV infections do not induce the expression of IFN-regulated genes, HBV is often described as a “stealthy” virus that is able to evade the host’s innate immune responses [34]. Nonetheless, recent studies have revealed that HBV infections reportedly trigger the innate immune responses through activation of the TLR2/myD88-dependent signaling pathway, which is rapidly controlled through negative feedback mediated by the HIPPO signaling pathway [11]. In the TLR/myD88-dependent signaling pathway, engagement of TLR leads to the formation of the Myddsome complex, which is comprised of the MyD88 and the IRAK kinase family [35]. The autophosphorylation and release of IRAK1 from the Myddsome, which ultimately leads to the activation of TAK1 [36]. Subsequently, the activation of TAK1 results in the activation of the IKK complex-NF-κB pathway and MAPK signaling pathway [37]. This results in the translocation of NF-κB to the nucleus, where it induces the expression of pro-inflammatory genes [37]. Likewise, the activation of TAK1 also leads to the activation of MAPK family kinases such as ERK1/2, P38, and JNK [37]. This activates down-stream AP-1 family transcription factors, which are involved in regulating of the host inflammatory response [38]. Based on our kinase activity analysis, several MAPK family kinases were predicted to have higher kinase activities after HBV infection. These include ERK1/2, p38, JNK, and the upstream MKK4/7 (Figure 4B). Furthermore, increase phosphorylation of STAT1 at Ser727, a known downstream effect of TLR/myD88 activation during the innate immune response, was also observed in our study (Supplementary Table S1 and Supplementary Figure S5D) [39]. While these observations suggest an activation of the TLR/MyD88-dependent signaling pathway, no activation of the HIPPO signaling pathway was observed. The dephosphorylation of YAP/TAZ1 transcription factors indicates the activation of the HIPPO signaling pathway [40]. However, no deregulation of phosphorylation was observed in all seven detected phosphosites in our study. This suggests that at seven dpi, HBV infection may not trigger the activation of the HIPPO signaling pathway. Based on these observations, our results suggest that the innate immune responses could be triggered during HBV infection at seven dpi through the activation of the myD88-dependent signaling pathway. However, further experimental evidence from immune cells will be needed to validate these results. 

To evaluate potential candidate kinases as drug targets, we tested several kinase inhibitors on HBV infection. MU1210, a highly potent inhibitors of CLK1, 2, and 4, exhibited significant suppression of HBV infection (Figure 5B). The CLK family kinases, consisting of dual specificity protein kinases CLK1, CLK2, CLK3, and CLK4, are highly conserved and found in diverse organisms ranging from yeast to humans [41]. CLKs are known to play critical roles in regulating mRNA splicing through the phosphorylation of SR proteins [42]. Our results indicate that CLKs could be involved in modulating HBV infection, but the exact role played by each of the CLKs during HBV infection should be delineated in subsequent studies. Although CLKs share homology with SRPK kinases, SRPK1 and CLKs phosphorylate distinct groups of proteins—as CLKs mainly localise to the nucleus while SRPK1 is cytoplasmic [43]. Therefore, it is unlikely that CLKs regulate HBV infection through the phosphorylation of HBcAg, a process that is crucial for viral RNA encapsidation. In other diseases such as prostate cancer and HIV infection, CLKs have been reported as promising therapeutic targets [44,45]. Although the biological significance of HBV RNA splice variants during infection is fragmented, the alternative splicing of pgRNA has been implicated in modulating HBV replication, which could lead to the translation of novel viral proteins that could alter HBV pathogenesis and the host immune responses [46]. Thus, we propose that the involvement of CLKs in HBV pgRNA alternative splicing might play an important role in regulating HBV infection. 

## 4. Materials and Methods

### 4.1. Cell Culture 

The human hepatoma cell line HepG2--NTCP, obtained from Dr Koichi Watashi (National Institute of Infectious Diseases, Tokyo, Japan) [47], was used for in vitro infection. The cells were maintained in complete medium consisting of Dulbecco’s modified Eagle’s medium (DMEM/F-12, GlutaMax supplement, Gibco, Waltham, MA, USA) supplemented with 5% heat inactivated fetal bovine serum (FBS) (Gibco, Waltham, MA, USA), 0.1 mM non-essential amino acids (NEAA) (Gibco, Waltham, MA, USA), 100 U/mL penicillin, 100 µg/mL Streptomycin (Gibco, Waltham, MA, USA), 2 mM L-glutamine (Gibco, Waltham, MA, USA), 5 µg/mL human insulin (Sigma Aldrich, St. Louis, MO, USA), and 500 µg/mL geneticin (G418) (Gibco, Waltham, MA, USA) at 37 °C in a humidified 5% CO_2_ incubator. 

### 4.2. HBV Infection

HBV were generated from a stable transfected hepablastoma cell line (Hep38.7) that expresses HBV as described previously [48]. Briefly, Hep38.7 (tet-off) cells were induced to produce HBV particles through tetracycline withdrawal. Twelve days after tetracycline removal, culture media containing HBV were harvested and filtered through a 0.22 µm polyethersulfone (PES) membrane filter (Corning, New York, NY, USA). The virus supernatant was then concentrated using a heparin column and eluted with high salt, followed by overnight dialysis in phosphate-buffered saline (PBS). Real-time quantitative PCR was used to quantify the genome copy number of the HBV DNA. For infection, HepG2-NTCP cells at 70% confluence 1-day post-seeding were first treated with synchronisation medium consisting of complete medium with 3% DMSO and 500 µM L-Mimosin for 24 h. the cells were then incubated for 30 min with complete medium containing 800 µM of EGTA at 37 °C in a humidified 5% CO_2_ incubator. After the removal of EGTA-containing medium, the cells were infected with HBV at a multiplicity of infection (MOI) of 3000 in the presence of 4% PEG 8000 and 2.5% DMSO in DMEM/F12 medium supplemented with 2% FBS for 24 h. For the mock infection, cells were incubated for 24-hour in 4% PEG 8000, 2.5% DMSO in DMEM/F12 medium supplemented with 2% FBS. Upon virus removal followed by washing with PBS four times, HBV-infected cells were cultured in post-infection medium consisting of DMEM/F12 medium with 2% FBS and 1% DMSO. The post-infection medium was replaced at four days post-infection (dpi) and infected cells were harvested at day seven dpi. For phosphoproteome analysis, five biological replicates each of mock and HBV-infected HepG2-NTCP were used.

### 4.3. Extraction of RNA and DNA

Total RNA was extracted using the RNeasy Mini Kit (Qiagen, Hilden, Germany) and digested with DNase according to the manufacturer’s protocol. For cDNA synthesis, 250–400 ng of total RNA was reverse transcribed with the iScript cDNA Synthesis Kit (Bio-Rad, Hercules, CA, USA) following the manufacturer’s protocol. cDNA was adjusted to 5 ng/µL using nuclease-free water and 2 μL of cDNA per reaction was used for qPCR analysis. Total DNA was extracted from the cells using the DNeasy Blood and Tissue Kit (Qiagen, Hilden, Germany) according to the manufacturer’s protocol.

### 4.4. Real-Time Quantitative PCR and HBeAg ELISA

To determine pgRNA level after HBV infection, HBV pgRNA was quantified by real-time qPCR using two HBV-specific primers: pgRNA-Forward, 5′-GTGCACTTCGCTTCACCTCT-3′, and pgRNA-Reverse, 5′-TTGACATTGCTGAGAGTCCAA-3′ [49]. The housekeeping gene, GAPDH, was amplified using GAPDH forward primer 5′-GTGTGAACCATGAGAAGTATGA-3′ and GAPDH reverse primer 5′-GTCCTTCCACGATACCAAAG-3′. The relative fold change of pgRNA between conditions was determined using the ΔΔct method. Real-time qPCR was performed with iTaq Universal SYBR Green Supermix (Bio-Rad, Hercules, CA, USA). To determine the copy number of intracellular cccDNA, a probe-based qPCR approach was used. cccDNA was amplified using cccDNA forward primer 5′-GGGGCGCACCTCTCTTTA-3′ and cccDNA reverse primer 5′-AGGCACAGCTTGGAGGC-3′. The Taqman probe 56, FAM/TCACCTCTG/ZEN/CCTAATCATCTC/3IABkFQ was used. The cccDNA copy number was determined using a standard curve approach with a 10× serial-diluted cccDNA template, starting from 10ng (Standard rang 1 fg to 1 ng). The reaction was carried out using the Luna Univeral Probe qPCR mix (NEB, Ipswich, MA, USA). To determine the level of HBeAg secretion, the HBeAg CLIA kit (Autobio diagnostic, Zhengzhou, China) was used. This assay was performed on 50 µL of medium harvested at four or seven dpi. All experiments were performed in triplicates and repeated twice for two additional biological replicates. The *p*-value was calculated using Student’s *t*-test (two-tailed, * *p*-value < 0.05, ** *p*-value < 0.01). 

### 4.5. Immunofluorescence

The cells on coverslips were fixed with 4% paraformaldehyde at room temperature (RT) for 15 min. After washing with PBS twice, the cells were permeabilised with 0.1% Triton X-100 in PBS for 15 min at RT. Following another PBS wash step, the cells were incubated with blocking buffer (1% BSA in PBS) for 30 min at RT. For HBcAg staining, the rabbit anti-HBcAg (DAKO 0586) primary antibody was diluted 1:500 in blocking buffer and incubated with the cells for 1 h at RT. The cells were then washed three times with blocking buffer before incubation with the secondary antibody (goat anti-rabbit IgG conjugated with Alexa Fluor 594 diluted 1:1000 in blocking buffer, Invitrogen) for 1 h in the dark at RT. After washing three times in PBS, the coverslips were mounted using the ProLong™ Diamond Antifade Mountant with DAPI (Invitrogen, Waltham, MA, USA) and imaged using EVOS FLoid Cell Imaging Station (Life Technologies, Carlsbad, CA, USA).

### 4.6. Cell Lysate Preparation

Cells harvested at seven dpi were lysed with lysis solution containing 9M urea, 20 mM HEPES, 1 mM sodium orthovanadate, 2.5 mM sodium pyrophosphate, and 1 mM β-glycerophosphate, which were supplemented with 1× Complete EDTA-free mini-protease inhibitor (Roche Diagnostic, Basel, Switzerland). Protein quantification was performed using the Pierce 660 nM Protein Assay (Thermo Fisher Scientific, Waltham, MA, USA) according to the manufacturer’s instructions. 

### 4.7. Reduction, Alkylation and Digestion

Lysate containing 500 µg total protein was first reduced with 5 mM dithiothreitol and was followed by alkylation with 10 mM iodoacetamide. The sample was then diluted to 6M urea with 100 mM ammonium bicarbonate before digestion with Lys-C protease (Wako, Osaka, Japan) at an enzyme-to-protein ratio of 1:100 (*w*/*w*) overnight at 37 °C. Subsequently, the sample was further diluted to 1M urea with 100 mM ammonium bicarbonate, before trypsin digestion at an enzyme-to-protein ratio of 1:50 (*w*/*w*) for 8 h at 37 °C. The resulting peptides were then desalted using the Empore C18-SD Extraction Disk Cartridge (3M, Saint Paul, MN, USA).

### 4.8. Tandem Mass Tag (TMT) Labelling

TMT10plex labelling was performed according to the manufacturer’s recommendation (Thermo Fisher Scientific, Waltham, MA, USA). Briefly, 100µg of digested peptides were labelled with each TMT reagent resuspended in 41 µL of anhydrous acetonitrile. The TMT-labelled peptides were mixed at equal ratios for a total of 1mg labelled peptide (Mock: 126, 127N, 127C, 128N, 128C; HBV infected; 129N, 129C, 130N, 130C and 131). The labelled peptide mixture was then dried using a vacuum concentrator before phosphopeptide enrichment.

### 4.9. Phosphopeptide Enrichment

Phosphopeptide enrichment was performed on the TMT-labelled peptide mixture using the Fe-NTA Phosphopeptide Enrichment Kit (Thermo Fisher Scientific, Waltham, MA, USA) based on the manufacturer’s instructions. Briefly, the lyophilised TMT-labelled peptides were resuspended with binding buffer before loading onto a spin column containing pre-equilibrated Fe-NTA. Enrichment was performed at RT for 30 min before washing three times with the washing buffer. Phosphopeptides were subsequently eluted from the beads and dried using a vacuum concentrator before resuspension with 0.1% formic acid. The peptide concentration was determined using the NanoDrop spectrophotometer (Thermo Fisher Scientific, Waltham, MA, USA). 

### 4.10. Mass Spectrometry Data Acquisition

Online reversed phase LC separation was performed on 4 µg of labelled-phosphopeptides using the EASY-nLC 1000 UPLC system (Thermo Fisher Scientific, Waltham, MA, USA). The enriched phosphopeptides were separated using an EASY-Spray column (C18, 100 Å, 2 µm, 75 µm × 500 mm, Thermo Fisher Scientific, Waltham, MA, USA) over a 180-min gradient (8–38% mobile phase B) at flow rate of 300 nL/min. Mobile phase A was 2% acetonitrile and 0.1% formic acid, and mobile phase B was 80% acetonitrile and 0.1% formic acid. The eluted peptides were directly injected into the Orbitrap Fusion Tribrid Mass Spectrometer (Thermo Fisher Scientific, Waltham, MA, USA) with the following parameters: spray voltage at 2.5 kV, RF lens level at 60%, and ion transfer tube temperature 275 °C. The full scan range of MS1 was defined as 400–1500 *m/z* at a resolution of 120K with a maximum ion injection time of 50 ms and automated gain control (AGC) target 4.0 × 10^5^ Data-dependent MS2 and MS3 (ddMS2 and ddMS3) were configured according to the MSA SPS MS3 method, as previously described [50]. Briefly, ddMs2 was performed using CID OT, with the collision energy at 35% and isolation width at 0.7 *m/z*. The maximum ion injection time was set at 60 ms with an Orbitrap resolution of 30 k and AGC target 5.0 × 10^4^. For the MS3 filter, the precursor selection was set to 400–1200 *m/z* with precursor ion exclusion ranges from −18 to +5. Isobaric tag loss exclusion was set to TMT. For ddMS3, HCD OT was used with an isolation width of 2 *m/z* and an HCD collision energy of 65%. The scan range was set to 100 to 500 *m/z* with a resolution of 60 K, ion injection time of 118 ms, and AGC target 1.0 × 10^5^. 

### 4.11. Identification and Quantification of Phosphopeptides

The generated MS spectra was searched using the Proteome Discoverer Software v2.2 (Thermo Fisher Scientific, Waltham, MA, USA). For phosphopeptide identification, the SEQUEST HT search engine was used. The search was performed using Percolator against the UniProt human reference proteomes database (June 2017 release, 92977 entries including isoforms), which was modified to include all the viral proteins of HBV genotype C and D (UniProt, 13 entries, accessed on May 2018) with an FDR specified at 0.01. The precursor and fragment mass tolerance were set at 10 ppm and 0.02 Da, respectively. The static modifications include cysteine carbamidomethylation (+57.021 Da) and TMT10plex tags on both the N-terminal and lysine (+229.163 Da). The dynamic modifications include methionine oxidation (+15.995 Da); serine, threonine, and tyrosine phosphorylation (+79.966 Da); and N-terminal acetylation (+42.011 Da). For confident identification of phosphorylated sites, the ptmRS node of PD2.2 was used for phosphosite localisation. Only phosphosites with a localisation probability of more than 90% were considered. Both unique and razor peptides were used for quantification. For reporter ion quantification, co-isolation threshold of 50% and average reporter signal-to-noise (S/N) threshold of 10 were used to consider peptides for quantification. The mass spectrometry proteomics data are deposited to the ProteomeXchange Consortium via the PRIDE [51] partner repository with the dataset identifier PXD015393 and DOI 10.6019/PXD015393. 

### 4.12. Analysis of Differentially Regulated Phosphoproteins

The identification and visualisation of differentially regulated phosphopeptides were performed using Perseus [52]. Phosphopeptides were considered differentially regulated when the fold change of HBV/Mock is >1.3 (up-regulated) and <0.769 (down-regulated), in addition to a *p-*value of <0.05, as determined by the Student’s *t*-test, after normality was determined using the Shapiro-Wilk test in R Studio version 1.3.1 (R Studio, Boston, MA, USA). Gene Ontology (GO) enrichment analysis and visualization of the relationships between ontology terms was performed using the ClueGO plugin in Cytoscape version v3.6.1 (Cytoscape Consortium) [17,53] with the following parameters: only GO terms annotating Biological Process (BP) or Immune System Process (ISP) was selected for analysis. Network Specificity of medium was chosen for plotting of the network, and a Kappa score for the GO terms and pathway network connectivity of more than 0.4 was chosen. A two-sided hypergeometric test was used to determine whether the terms are enriched or depleted, and an adjusted *p-*value < 0.05 was considered significant. 

### 4.13. Kinase–Substrate Prediction and Enrichment Assay

Prediction of upstream kinases was performed using NetworKIN (v3.0) based on the kinase consensus motifs and their cellular context [54]. Phosphosites from all differentially regulated phosphopeptides were used for upstream kinase prediction. the default Networkin score of 1.5 was chosen as the cut-off for the prediction. Kinase–substrate enrichment assay (KSEA) was performed to predict the activity of kinases with the delta count approach [14]. Briefly, with the NetworKIN prediction output, the sample frequency of kinases was calculated based on the number of times the kinase–substrate pair was predicted. By comparing the kinase sample frequency with the kinase population frequency, which was calculated based on the Networkin prediction of the whole human proteome, the activity of kinases can be estimated with two-sided hypogeometric testing. 

### 4.14. Cytotoxicity Assay

The IC_50_ of kinase inhibitors was determined using the Cell-titer-GLO Luminescent Cell Viability Assay kit (Promega, Madison, WI, USA) according to the recommendations from the manufacturer. Briefly, 10,000 HepG2-NTCP cells were seeded into a 96-well plate before treatment with various kinase inhibitor concentrations. Cell viability was assayed at 24 h post-treatment. 

### 4.15. Kinase Inhibitor Treatment of HBV Infected HepG2-NTCP

HBV infection on HepG2-NTCP was carried out as described earlier. At one dpi, the kinase inhibitors (Table 1) were added to the post-infection medium after the washing and removal of viruses containing infection medium. For CK2 inhibition, 10 µM of DMAT (SML2044, Sigma-Aldrich, St. Louis, MO, USA) was used. For CDK1 inhibition, 100 nM of RO-3306 (SML0569, Sigma-Aldrich, St. Louis, MO, USA) was used. For CDK2 and CDK5 inhibition, 5 µM of PNU112455A (SML0498, Sigma-Aldrich, St. Louis, MO, USA) was used. For CLKs inhibition, 1 µM of MU1210 (SML2713, Sigma-Aldrich, St. Louis, MO, USA) was used. In addition, 1 µM of MU140 (SML2722, Sigma-Aldrich, St. Louis, MO, USA), a structural analogue, was used as a control for MU1210. At four dpi, the expended medium was replaced with fresh post-infection medium containing the corresponding kinase inhibitors. Infected HepG2-NTCP was harvested at four and seven dpi for pgRNA quantification.

## 5. Conclusions

Our unbiased advanced phosphoproteomics analyses provide a detailed snapshot of the phosphoproteome changes in hepatocytes during HBV infection. Subsequent data-driven bioinformatics analyses revealed the dynamics in the host kinome and phosphosignaling events impacted by HBV. These dynamic events should be further studied to confirm and assess the viability of their key hubs as targets for HBV therapeutics. Of note, our comprehensive study will be a valuable resource to explore subsequent mechanistic details on the role of protein phosphorylation in regulating HBV pathogenesis, and key signaling proteins with aberrant activities that could be potential entry points in HBV drug target discovery.

## Figures and Tables

**Table 1:** Information on selected kinase inhibitors.

Kinases Inhibitor	Target	Reported Ki	IC_50_
DMAT	CK2	40 nM	>500 µM
RO-3306	CDK1	35 nM	164 µM
PNU112455A11	CDK2 and CDK5	3.2/3.6 µM (CDK2/5)	>500 µM
MU1210	CLKs	23 nM	16.7 mM
MU140	NA (Structural analog of NU1210)	NA	>5 00 µM

## Data Availability

Data are available via ProteomeXchange with identifier PXD015393, project DOI: 10.6019/PXD015393.

## Supplementary Materials

The following supporting information can be downloaded at: https://www.mdpi.com/article/10.3390/ijms23095127/s1.
