# Supplementary material for: Phosphoproteomics Unravel HBV Triggered Rewiring of Host Phosphosignaling Events
Source: Int J Mol Sci. 2022 May 4;23(9):5127. doi: 10.3390/ijms23095127 (PMC9104152; doi:10.3390/ijms23095127)
Supplement: Supplementary file 1 [file ijms-23-05127-s001.zip › supplementary Information.pdf]

Supporting information

Supplementary Figure S1. Immunofluorescence staining of HBcAg of 7dpi HBV infected HepG2-NTCP

Supplementary Figure S2. (A) PCA and (B) Unsupervised clustering of HepG2-NTCP phosphoproteome during HBV infection

Supplementary Figure S3. GO-BP analysis of down-regulated phosphoproteins.

Supplementary Figure S4. Significance map of ClueGo network of up-regulated phosphoproteins.

Supplementary Figure S5. GO analysis restricted to immune system processes. (A) ClueGO analysis of proteins with dysregulated phosphorylation focusing on Immune System Process (ISP). (B) Depicting of ClueGO map based of significance (*p-value*). (C) Top ISP GO terms associated with dysregulated phosphoproteins. (D) Heatmap (z-score) depicting abundance changes of phosphoproteins associated with ISP GO-terms.

Supplementary Figure S6. MS/MS spectra of novel phosphosite SRPK1 ser482

Supplementary Figure S7. Chemical cytotoxicity assay of Kinase inhibitors with detectable toxicity. Cytotoxicity assay was performed on HepG2-NTCP with Ro-3306, MU1210, MU140, PNU112455A and DMAT. Under the condition tested, cytotoxic effect was only observed when HepG2-NTCP was treated with Ro-3306 (IC_{50}: 164 nM) and MU1210 (IC_{50}:16.7 µM). For the other inhibitors, the cells retained 100% viability even at the highest concentration used based on solubility of drugs.

Supplementary Figure S8. Effects of kinase inhibitors on pgRNA transcription in HBV infected HepG2-NTCP. HepG2-NTCP was treated with 10 µM of DMAT, 100 nM of Ro-3306, 5 µM of PNU112455A and 1 µM of MU1210 (MU140 structural analogue as control). Results showed here were based on three biological replicates.

Supplementary Table S1. Phosphosites of host proteins deregulated at 7dpi

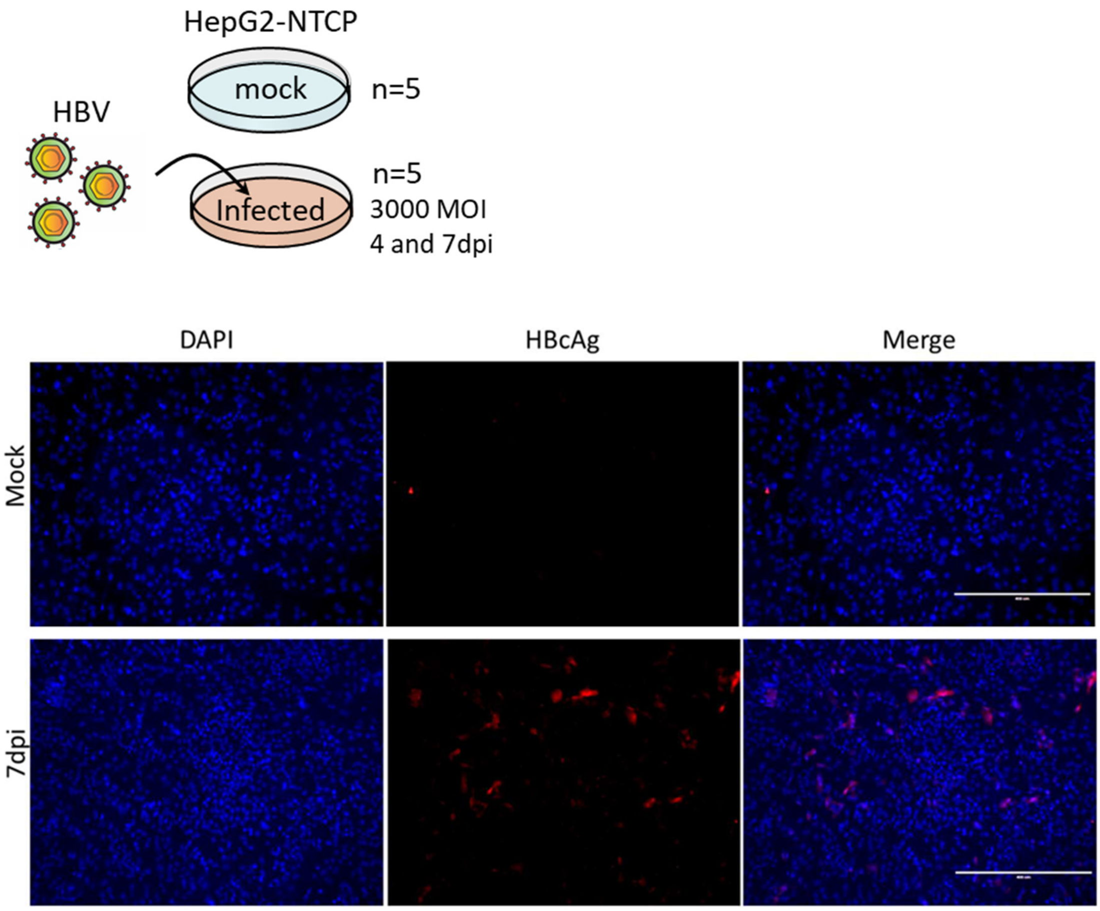

Supplementary Figure S1. Immunofluorescence staining of HBcAg of 7dpi HBV infected HepG2-NTCP.

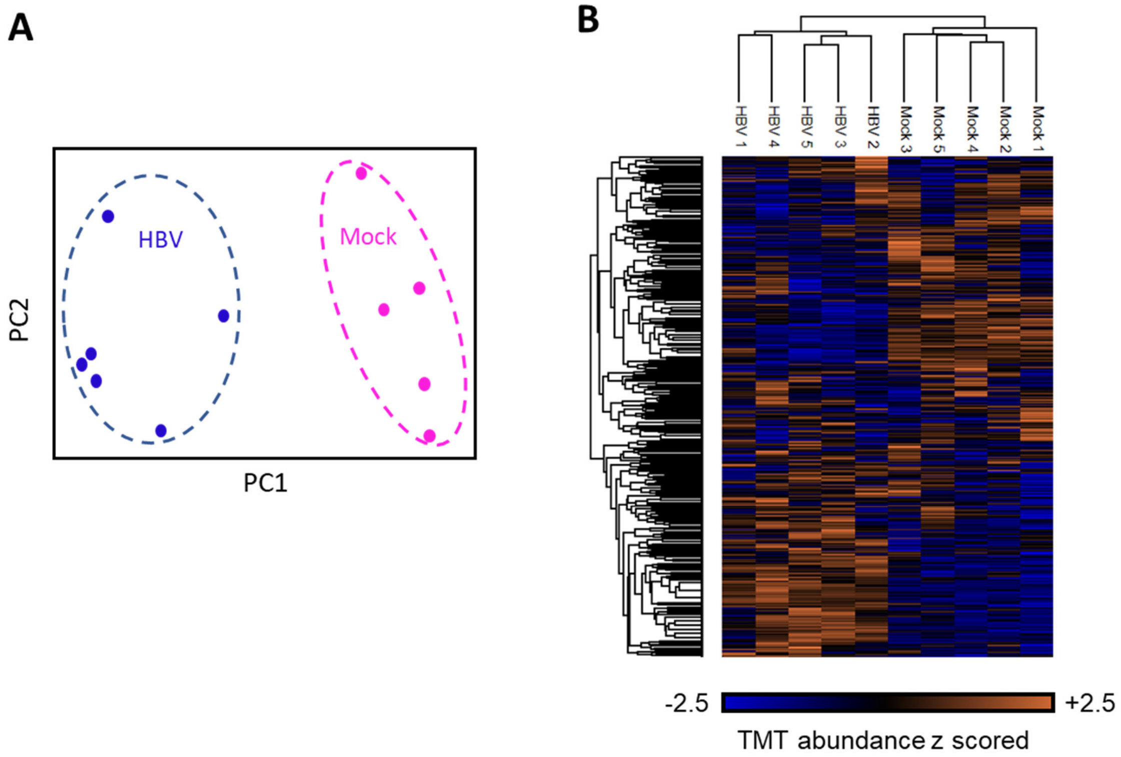

Supplementary Figure S2. (A) PCA and (B) Unsupervised clustering of HepG2-NTCP phosphoproteome during HBV infection.

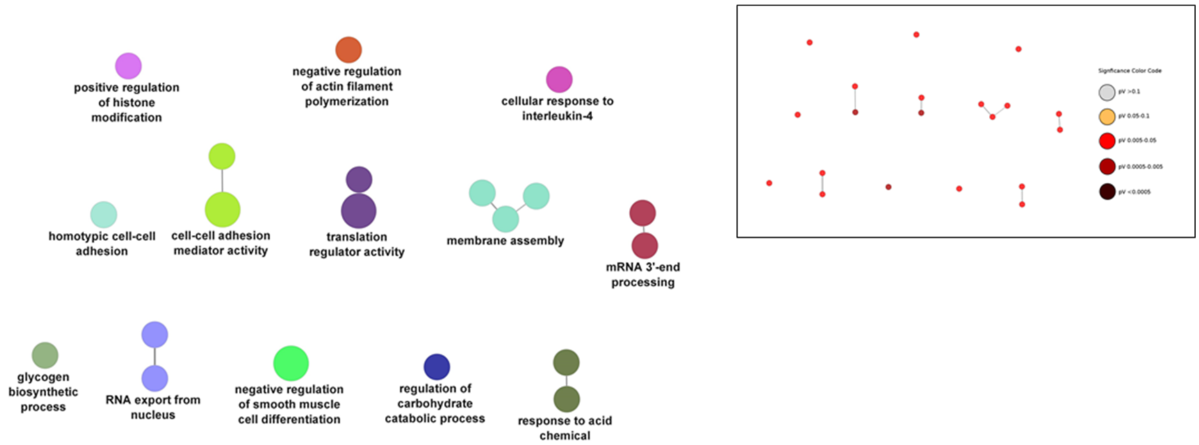

Supplementary Figure S3. GO-BP analysis of down-regulated phosphoproteins.

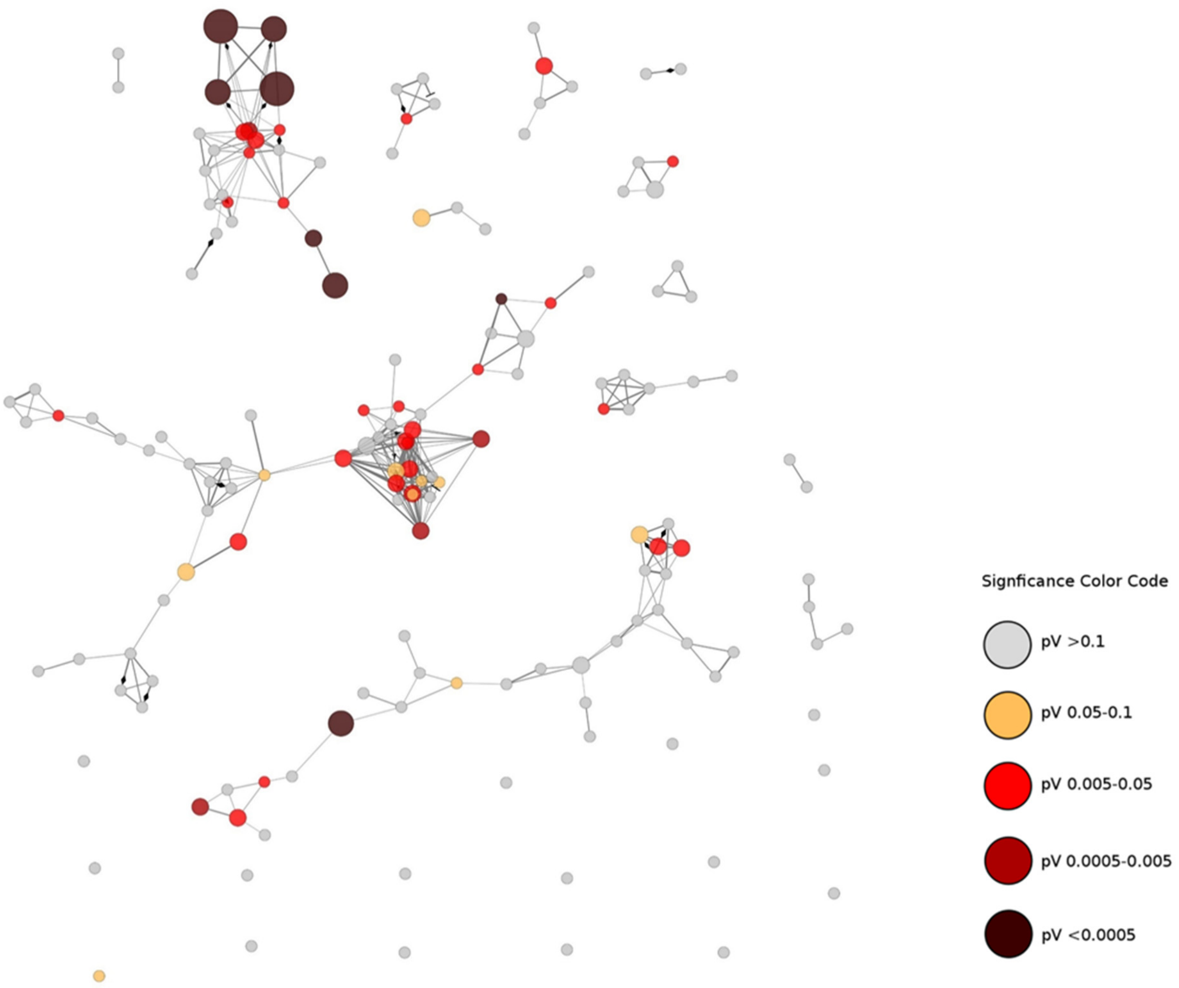

Supplementary Figure S4. Significance map of ClueGo network of up-regulated phosphoproteins.

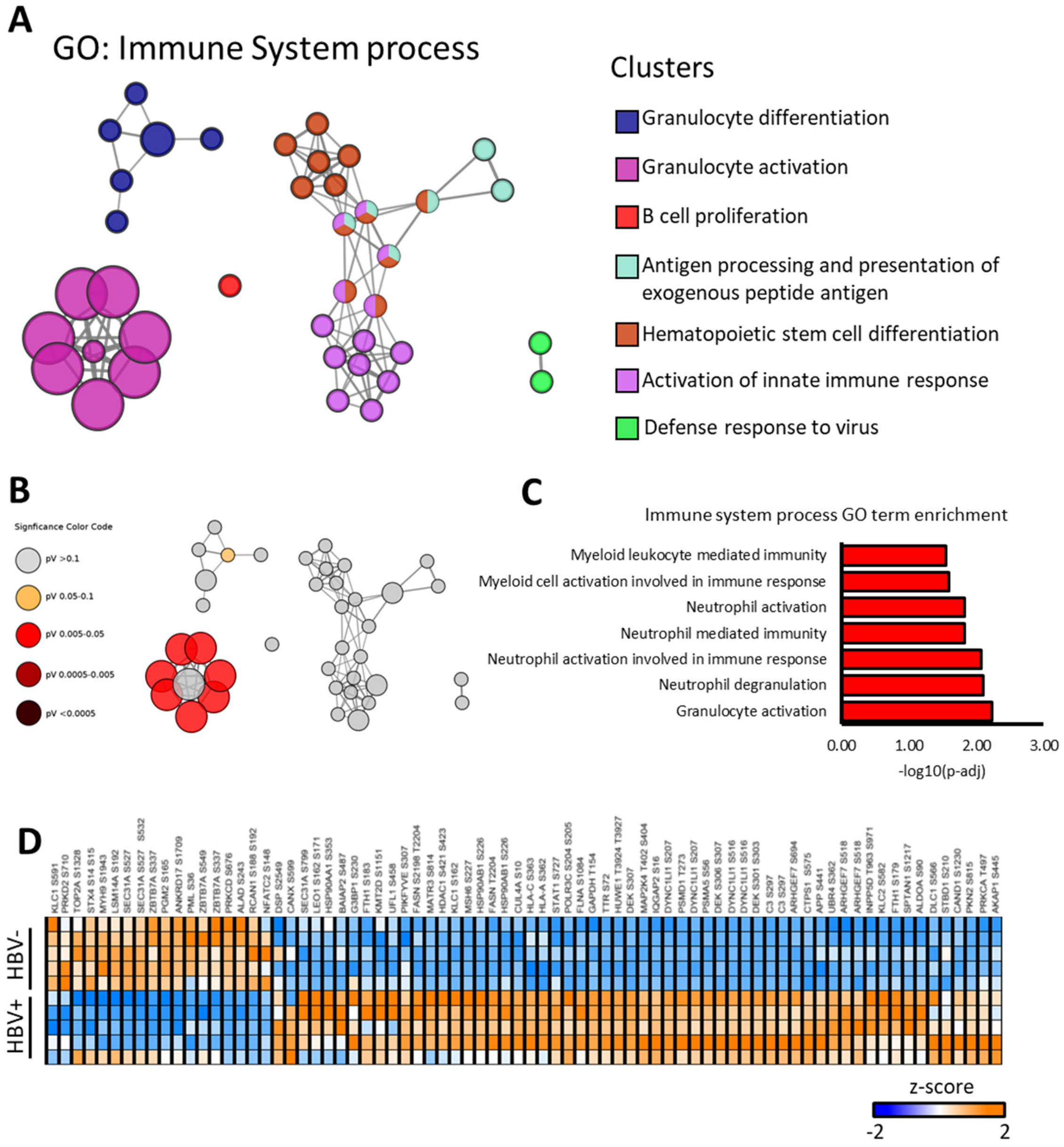

Supplementary Figure S5. GO analysis restricted to immune system processes. (A) ClueGO analysis of proteins with dysregulated phosphorylation focusing on Immune System Process (ISP). (B) Depicting of ClueGO map based of significance (*p-value*). (C) Top ISP GO terms associated with dysregulated phosphoproteins. (D) Heatmap (z-score) depicting abundance changes of phosphoproteins associated with ISP GO-terms.

#1	b+	b2+	b3+	Seq.	y+	y2+	y3+	#2
1	358.22879	179.61803	120.08111	Q-TMT6plex				13
2	487.27138	244.13933	163.09531	E	1708.83561	854.92144	570.28339	12
3	616.31397	308.66062	206.10951	E	1579.79302	790.40015	527.26919	11
4	703.34600	352.17664	235.12018	S	1450.75043	725.87885	484.25499	10
5	832.38859	416.69794	278.13438	E	1363.71840	682.36284	455.24432	9
6	999.38695	500.19711	333.80050	S-Phospho	1234.67580	617.84154	412.23012	8
7	1096.43972	548.72350	366.15142	P	1067.67745	534.34236	356.56400	7
8	1195.50813	598.25770	399.17423	V	970.62468	485.81598	324.21308	6
9	1324.55072	662.77900	442.18843	E	871.55627	436.28177	291.19027	5
10	1480.65183	740.82956	494.22213	R	742.51367	371.76048	248.17608	4

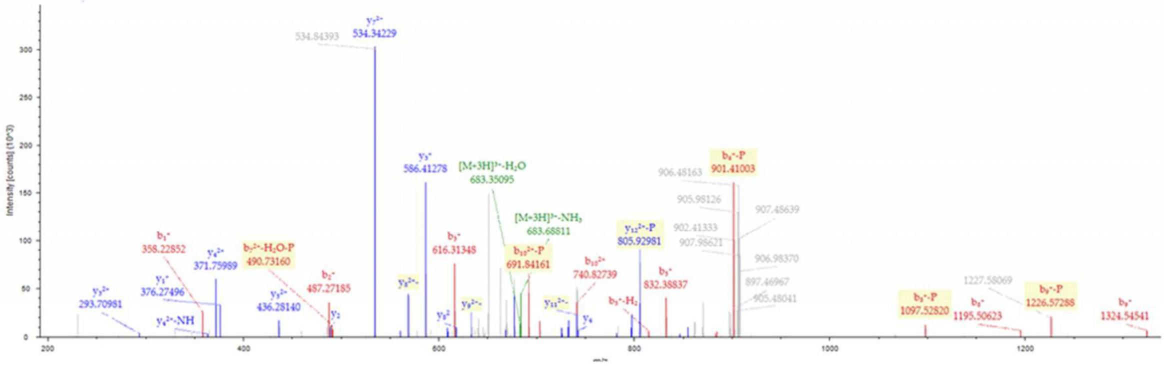

Supplementary Figure S6. MS/MS spectra of novel phosphosite SRPK1 ser482.

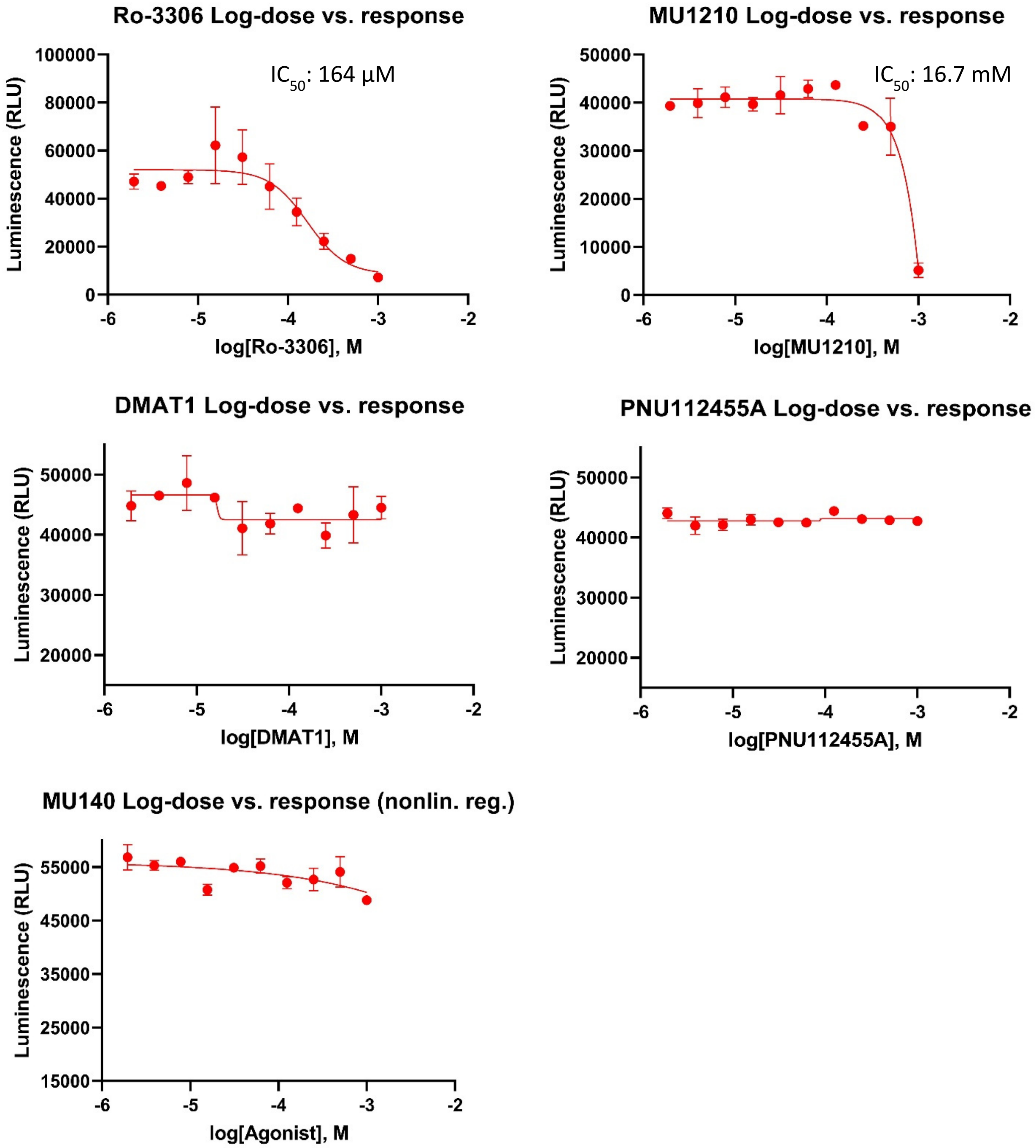

Supplementary Figure S7. Chemical cytotoxicity assay of Kinase inhibitors with detectable toxicity. Cytotoxicity assay was performed on HepG2-NTCP with Ro-3306, MU1210, MU140, PNU112455A and DMAT. Under the condition tested, cytotoxic effect was only observed when HepG2-NTCP was treated with Ro-3306 (IC_{50}: 164 nM) and MU1210 (IC_{50}:16.7 µM). For the other inhibitors, the cells retained 100% viability even at the highest concentration used based on solubility of drugs.

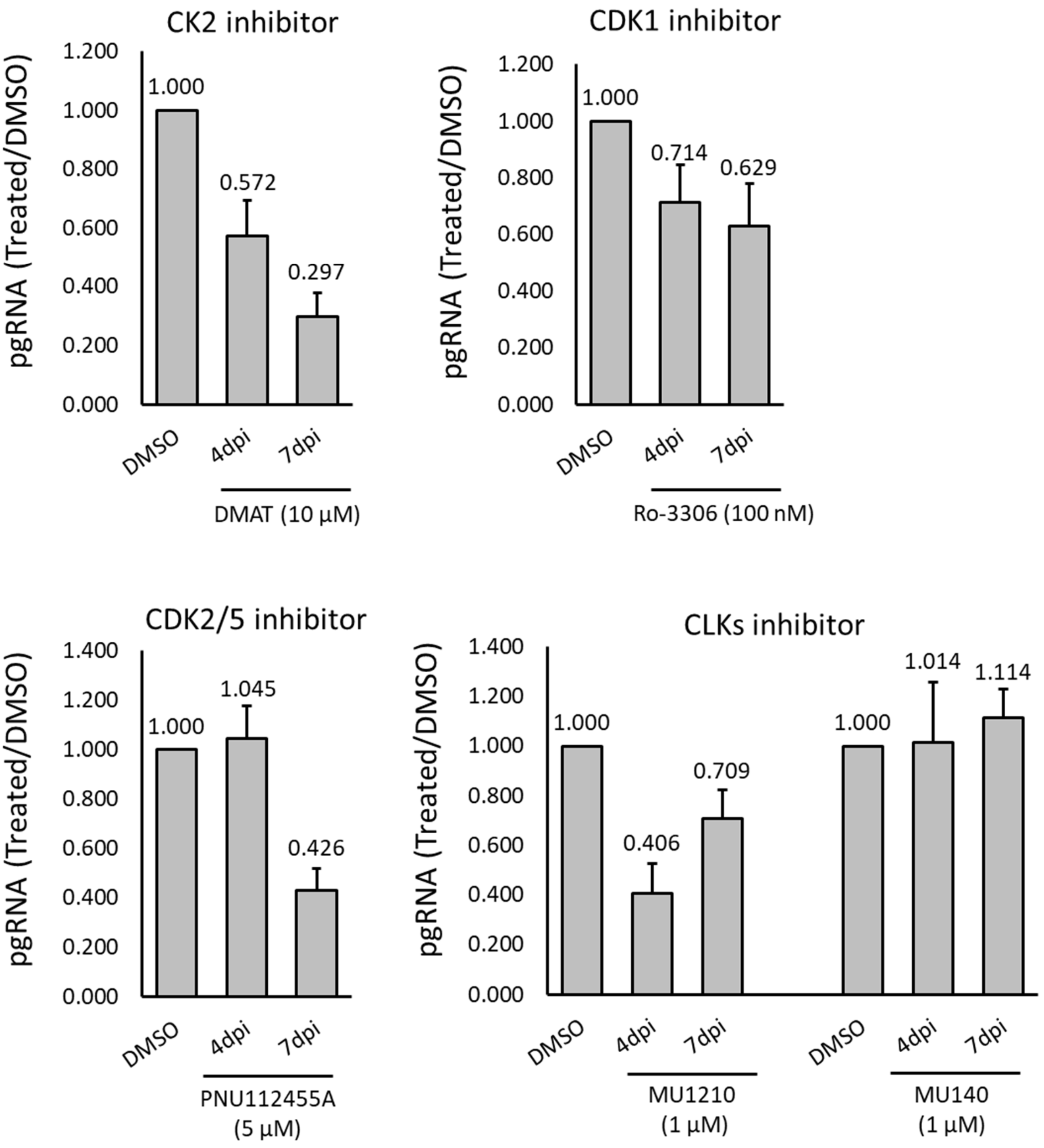

Supplementary Figure S8. Effects of kinase inhibitors on pgRNA transcription in HBV infected HepG2-NTCP. HepG2-NTCP was treated with 10 µM of DMAT, 100 nM of Ro-3306, 5 µM of PNU112455A and 1 µM of MU1210 (MU140 structural analogue as control). Results showed here were based on three biological replicates.
